# The Behaviour of Contaflex Soft Contact Lens Material During Hydration

**DOI:** 10.3390/gels11050376

**Published:** 2025-05-21

**Authors:** Joseph Towler, Markus Zaplachinski, Roberto Montiel, Nikhil Murari, Christine Deng, Rimmo Lego, Arwa Fathy, Ahmed Abass

**Affiliations:** 1 Department of Eye and Vision, Institute of Life Course and Medical Sciences, University of Liverpool, Liverpool L7 8TX, UK; 2 Department of Materials, Design and Manufacturing Engineering, School of Engineering, University of Liverpool, Liverpool L69 3GH, UK; 3 Department of Biomedical Engineering, Schulich School of Engineering, University of Calgary, Calgary, AB T2N 1N4, Canada; 4 School of Environmental, Civil, Agricultural, and Mechanical Engineering, University of Georgia, Athens, GA 30602, USA; 5 Department of Chemical, Materials & Biomedical Engineering, College of Engineering, University of Georgia, Athens, GA 30602, USA; 6 Department of Microbiology & Immunology, Schulich School of Medicine & Dentistry, Western University, London, ON N6A 5C1, Canada; 7 Department of Biomedical Engineering, Scaefer School of Engineering, Stevens Institute of Technology, Hoboken, NJ 07030, USA; 8 Department of Engineering, University of Cambridge, Cambridge CB2 1PZ, UK

**Keywords:** hydrogel, soft, contact lens, material, hydration, design, manufacturing

## Abstract

The aim of this study was to quantitatively evaluate the swelling and transparency behaviour of Contaflex soft contact lens materials with varying water-content (38–77%) using high-resolution digital imaging and infrared LiDAR. Contaflex materials with 38%, 55%, 58%, 67% and 77% nominal water-contents, denoted as C38, C55, C58, C67, and C77, were tested. Hydrogel samples (N = 5 per group) were monitored over 24 h in pH 7.1 phosphate-buffered saline. Dimensional changes were assessed via linear and radial expansion factors (LEF and REF), and transparency was tracked during hydration. All groups exhibited rapid initial swelling followed by continued expansion. LEF and REF values increased with water-content; C77 reached LEF and REF values of 1.563 ± 0.093 and 1.536 ± 0.052, while C38 stabilised near 1.201 ± 0.019 and 1.179 ± 0.011, respectively. Refractive index decreased with hydration, from 1.552 in C38 to 1.372 in C77. Power simulations revealed deviations beyond ISO tolerance limits in most materials, particularly those with higher water-content. Transparency changes were consistent with swelling dynamics. These findings support the need for material-specific design adjustments to account for hydration-related dimensional and optical changes in soft contact lenses.

## 1. Introduction

Hygroscopic properties are critical characteristics of hydrogels used in soft contact lens manufacturing, playing a pivotal role in lens performance and user comfort. These hydrogels exhibit both solid and liquid attributes; the polymer network structure contributes to the solid behaviour of the hydrogel, while its capacity for absorbing solvents enables liquid-like transport of matter within the lens [1]. This dual functionality supports essential moisture exchange, influencing hydration dynamics crucial for maintaining ocular health during wear [2,3].

Various factors, including material composition, environmental conditions, and the presence of ophthalmic solutions, can influence the hydration behaviour of hydrogel contact lenses [4]. Key industrial unitless metrics in evaluating hydrogel swelling for contact lenses are the linear expansion factor (LEF), which quantifies the material’s change in diameter (edge to edge) and the radial expansion factor (REF), which quantifies the material’s change in sagittal depth upon hydration. LEF and REF estimation accuracies are essential for manufacturing and clinically meaningful, impacting fit, on-eye stability, and comfort for contact lens wearers [5].

Measurement techniques for assessing hydration behaviour vary, including hand refractometry, gravimetric analysis, and low-field nuclear magnetic resonance [6,7]. These tools are crucial for understanding hydration states and ensuring the clinical performance of lenses. Understanding these interactions allows for better lens design and improved patient outcomes, aligning with the necessity of maintaining optimal hydration for comfort and reducing risks like corneal erosion [6].

While numerous studies have examined hydrogel lens behaviour, most focus on individual properties such as water-content, swelling ratios, or refractive index in isolation [6,8,9]. Gulsen and Chauhan, for example, investigated transparency and swelling in poly hydroxyethyl methacrylate (pHEMA) gels under equilibrium conditions but did not link these to dimensional changes or optical functions [8]. Similarly, González-Méijome et al. and Efron & Morgan characterised refractive index and equilibrium water-content relationships in conventional and silicone hydrogel lenses without addressing their dynamic behaviour during hydration [6,9].

Recent work has used real-time imaging techniques to evaluate in situ dimensional changes. Mlyniuk et al. employed optical coherence tomography (OCT) to track curvature and diameter shifts in daily disposable lenses after wear [10] and reported similar on-eye swelling behaviour during hypoxic conditions. However, these studies did not assess optical clarity or model refractive performance shifts.

Very few studies have attempted to monitor transparency and dimensional changes during hydration concurrently or to relate these changes directly to optical performance metrics. As a result, there is a lack of integrated, time-resolved data describing how the swelling process influences key soft contact lens parameters such as sagittal depth and power accuracy. The present study addresses this gap by combining high-resolution image tracking with light detection and ranging (LiDAR)-based optical transparency assessment, and cutting-edge refractive error simulation across a range of commercial hydrogel materials.

This study aims to quantitatively evaluate the swelling and optical transparency behaviour of group-I polymacon hydrogels at nominal water-contents ranging from 38% to 77%, as described by the manufacturer (Contamac Ltd., Shire Hill, Saffron Walden, Essex, UK). Using phosphate-buffered saline (PBS) (Sigma Aldrich, Dorset, UK), as the solvent, better simulates the ocular environment, allowing hydrogel dimensions to be tracked and observing LEF and REF changes over time. Our findings compare supplier-published technical data with measured outcomes, providing insights that can enhance contact lens material selection, design, manufacturing and, ultimately, patient comfort and lens optical performance.

## 2. Results and Discussion

### 2.1. Results

The results presented in this study highlight the hydration behaviour of Contaflex hydrogel materials with varying nominal water-content, ranging from 38% to 77%. Detailed analysis was conducted using LEF and REF measurements obtained through high-resolution digital image processing over two key time points, 17 h and 24 h, Table 1. Statistical comparisons, including paired t-tests and Wilcoxon signed-rank tests, were employed to assess differences across sample groups and time points. Furthermore, transparency changes during the hydration process were monitored using a custom-built LiDAR system, providing insights into the relationship between hydration levels and optical properties of the materials. Additionally, the study explored the implications of swelling behaviours on the accuracy of optical power in soft contact lens design through state-of-the-art computer simulation.

#### 2.1.1. Linear Expansion Factor (LEF)

**Figure 1:**
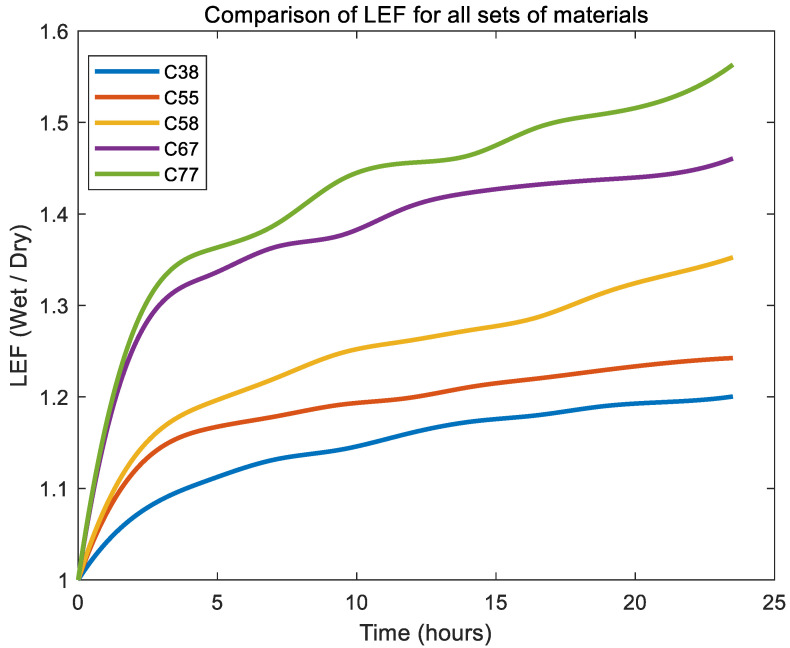
Comparison of mean LEF for Contaflex sets of materials.

All material groups showed an initial rapid increase in LEF, indicating rapid swelling during the early hydration stages within approximately the first 8 h, followed by a plateau or gradual stabilisation trend. Figure 1 illustrates the LEF, expressed as the ratio of the wet-to-dry dimensions, for Contaflex hydrogels with varying nominal water-content (C38, C55, C58, C67, and C77) over a 24 h hydration period. Samples were named with “C” indicating Contaflex; the number is the manufacturer’s stated water-contents.

Hydrogel group C77 exhibited the highest LEF, reaching approximately 1.56, which suggests this material exhibits the most pronounced swelling and remains in a transient hydration phase for longer, taking more time to reach a relatively steady-state condition compared to other hydrogel groups. Conversely, the C38 group demonstrated the lowest LEF values, stabilising near 1.2, reflecting the least swelling and highest dimensional stability. Intermediate groups (C55, C58, C67) displayed progressively increasing LEF values consistent with their respective water-content, with C67 reaching around 1.45 and C55 around 1.22 after 24 h.

A statistical analysis was conducted to evaluate differences in LEF values between the C38 and C58 sample groups at the 24 h time point (Table 2). A two-sample t-test revealed a significant difference in LEF between these two groups (*p* = 2.51 × 10^−7^), indicating that the observed variation in swelling behaviour was unlikely due to random chance.

**Figure 2:**
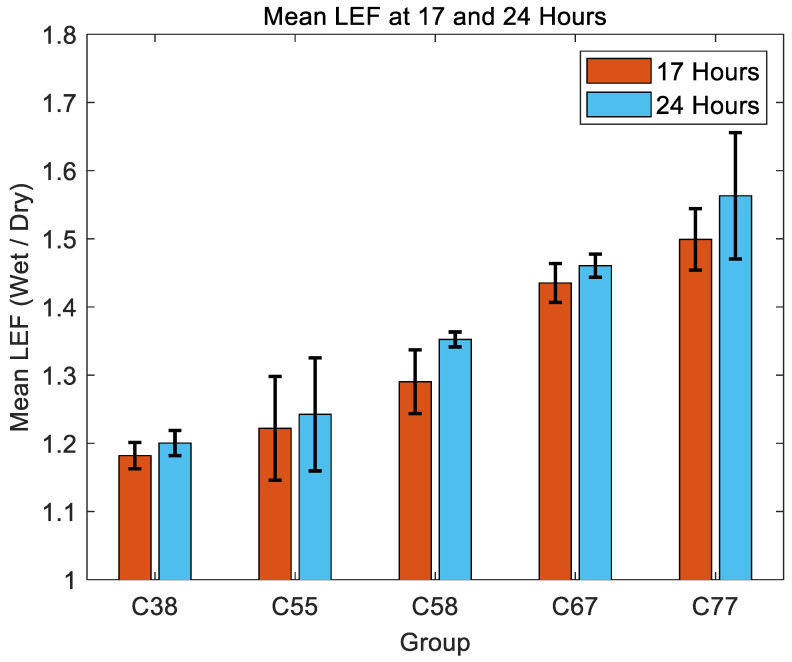
Mean LEF at 17 h and 24 h for all groups with error bars representing standard deviation.

Figure 2 compares the mean LEF values at 17 h and 24 h, the two key hydration points, for Contaflex hydrogels of different nominal water-contents (C38, C55, C58, C67, and C77). Across all material groups, the mean LEF increased between 17 h and 24 h, indicating an ongoing slow rate of material swelling over this interval. The increase in LEF was more prominent in higher water-content materials. Specifically, the C77 group exhibited the greatest swelling, with mean LEF values increasing significantly from approximately 1.5 at 17 h to 1.56 at 24 h. Conversely, the C38 group showed minimal swelling, growing slightly from about 1.18 at 17 h to 1.2 at 24 h, demonstrating greater dimensional stability. Intermediate materials (C55, C58, and C67) displayed moderate increases in swelling between the two measured time points, with mean LEF values progressively higher as water-content increased. Standard deviation bars indicate notable variability within groups, particularly in higher water-content groups, reflecting greater variation in their swelling behaviour.

A Wilcoxon signed-rank test was conducted to determine whether there was a significant difference in LEF area under the curve (AUC) values between 17 h and 24 h. The test produced a *p*-value of 0.0625, indicating that while there is some evidence of a difference, it does not reach conventional statistical significance (*p* ≤ 0.05).

A Hodges–Lehmann (HL) estimator was used to compute the median change in LEF AUC between the two time points to quantify the magnitude of the observed differences. The estimated median difference for each group is presented in Table 1. In all cases, the HL estimate was negative, confirming that AUC at 24 h was consistently larger than at 17 h. The 95% confidence intervals do not include zero, suggesting that the observed changes are unlikely to be due to random variation (Table 2).

#### 2.1.2. Radial Expansion Factor (REF)

**Figure 3:**
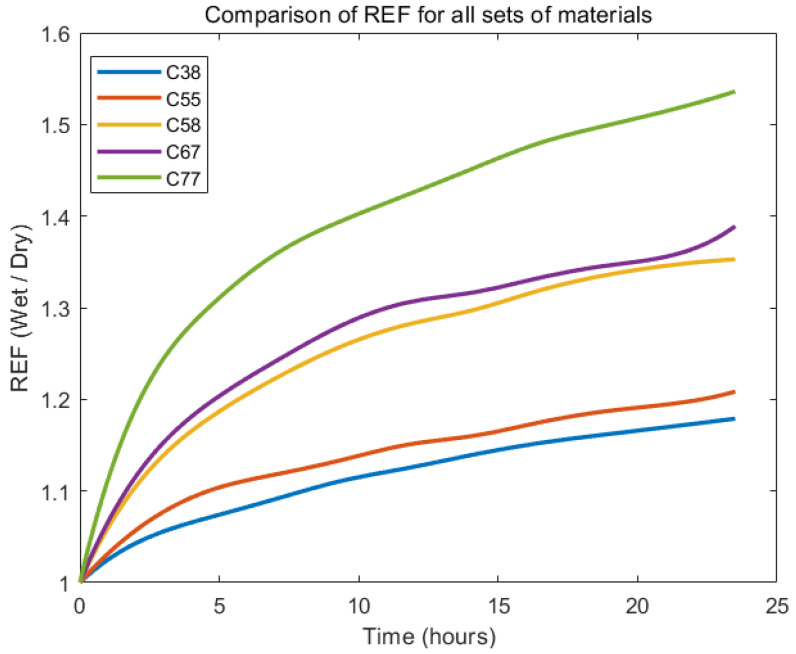
Comparison of the mean REF for all Contaflex groups.

All material groups exhibited a relatively rapid initial increase in REF, reflecting substantial radial swelling in the early hydration stages (within approximately the first 8 h). Subsequently, REF growth gradually slowed but continued steadily up to the 24 h mark. Figure 3 illustrates the REF of Contaflex hydrogels with different nominal water-contents (C38, C55, C58, C67, C77) across a 24 h hydration period.

Materials with higher water-content consistently showed greater radial swelling. Specifically, the C77 group demonstrated the highest overall swelling, approaching a REF value of around 1.5 after 24 h, whereas the C38 group showed the least swelling, stabilising near a REF of approximately 1.18. Intermediate groups (C55, C58, C67) displayed proportional swelling with increasing water-content, achieving REF values around 1.21, 1.35, and 1.39, respectively, after 24 h.

**Figure 4:**
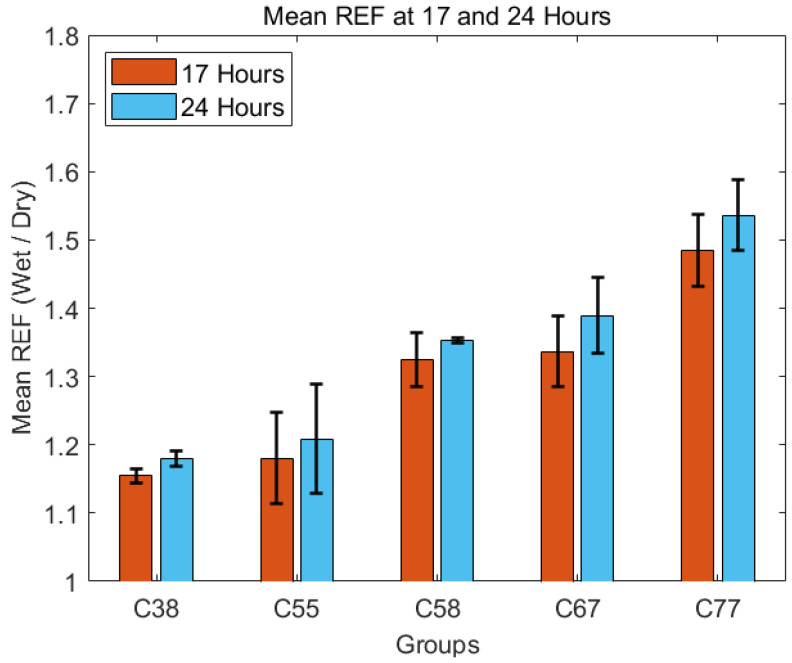
Mean REF at 17 h and 24 h for all groups with error bars representing standard deviation.

REF values increased between 17 h and 24 h for all groups, with higher water-content hydrogels demonstrating more swelling. Figure 4 compares mean REF values for various Contaflex hydrogel groups (C38, C55, C58, C67, and C77) at two distinct hydration time points, 17 h and 24 h.

Specifically, the C77 group exhibited the highest REF values, increasing from approximately 1.47 at 17 h to around 1.53 at 24 h. The C67 group followed a similar but slightly lower swelling profile, with REF values rising from 1.33 to 1.39. The lower water-content hydrogels (C38 and C55) showed more moderate increases, with C38 changing from about 1.16 to 1.18 and C55 from roughly 1.19 to 1.21. The standard deviation error bars indicate greater variability within higher water-content groups, suggesting less uniform swelling characteristics than lower water-content materials.

A Wilcoxon signed-rank test was also conducted for the REF AUC values to assess potential differences between 17 h and 24 h. Similar to the LEF dataset, the test returned a *p*-value of 0.0625, which suggests a trend toward a difference but does not meet conventional statistical significance (*p* = 0.05).

The HL estimator was again used to quantify the magnitude of the observed changes, with results presented in Table 2. Across all groups, the HL estimate was negative, confirming that AUC at 24 h was consistently larger than at 17 h. The 95% confidence intervals (CI) do not include zero, supporting the likelihood that this increase represents a true effect rather than random variation (Table 3).

#### 2.1.3. Refractive Indices

The refractive indices were measured to observe how light bends when passing through different materials. The results aligned with Snell’s law, showing the relationship between the angles of incidence and refraction.

Table 4 shows that the refractive index decreased with increasing water-content: C38 samples exhibited the highest values, followed by C55 and C58. Across all valid measurements, refractive index values ranged from approximately 1.38 to 1.52, consistent within each material group and demonstrating a clear inverse relationship between water-content and optical density.

#### 2.1.4. Soft Contact Lens Performance Simulation

**Figure 5:**
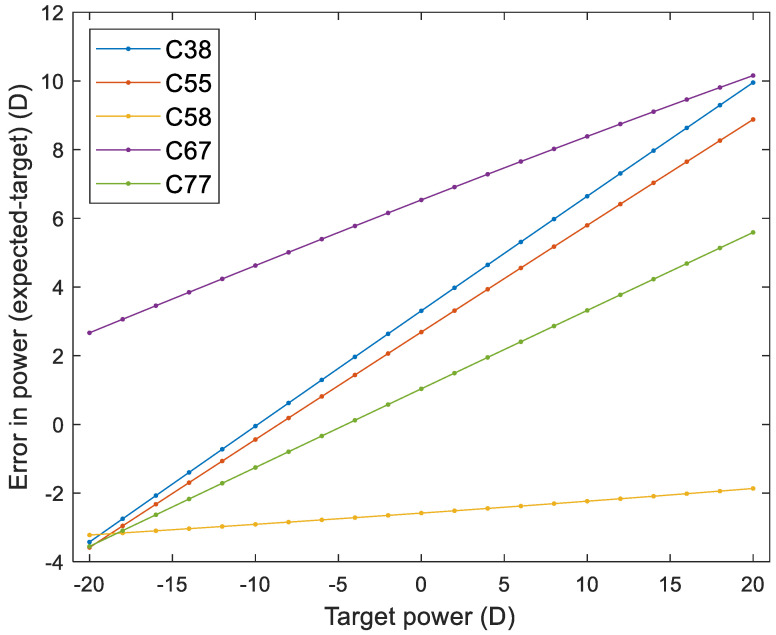
Power error as the difference between the expected and target power.

Figure 5 illustrates the optical power error, defined as the difference between the expected and target powers, across different Contaflex materials (C38, C55, C58, C67, and C77) over a range of target optical powers from −20 D to 20 D. The results demonstrate distinct material-specific trends. Material C38 demonstrated the steepest error gradient among all samples, with power deviations rising linearly from approximately −4 D to 10 D across the tested range.

Similarly, materials C55 and C67 followed strong positive error gradients, displaying consistent linear error growth with increasing target powers. C55 reached deviations of around ±9 D, while C67 climbed slightly higher, up to ±10 D. Both materials exhibited symmetrical behaviour around the zero target point.

In contrast, material C77 presented a more moderate error gradient. Though errors increased with target power, the slope was gentler, resulting in a total deviation of just under ±6 D at the extremes.

Material C58 stood out distinctly, exhibiting the flattest gradient of all. Its error curve remained relatively flat, with only a slight upward trend across the range, staying close to −2 D regardless of the target power.

Notably, the errors observed in these materials far exceed the tolerance thresholds outlined by the International Organisation for Standardisation (ISO): ±0.25 D up to ±10 D, increasing to ±0.5 D for powers up to ±20 D, and extending to ±1.0 D beyond that. Therefore, correction strategies are essential for clinical acceptance.

#### 2.1.5. Water-Content

In order to quantify the swelling response of each sample group, the mean mass swelling was calculated as the ratio of the mass increase to the initial mass. The results are summarised in Table 5.

C77 exhibited the highest moisture uptake among all groups, with a mean mass swelling of 0.637 ± 0.134, followed by C67 (0.607 ± 0.024) and C58 (0.522 ± 0.053). The lower swelling values were observed in C55 (0.331 ± 0.156) and C38 (0.265 ± 0.128), indicating reduced moisture absorption compared to the other groups.

The standard deviation values suggest some variability within each group, particularly in C55, which exhibited the highest variation (±0.156), whereas C67 had the lowest variability (±0.024), indicating more consistent swelling behaviour within that sample group.

These findings indicate a progressive increase in moisture uptake from C38 to C77, suggesting that higher sample indices are associated with greater swelling capacity. This trend aligns with the LEF and REF values, where swelling behaviour significantly changes over time.

#### 2.1.6. Transparency Detection

**Figure 6:**
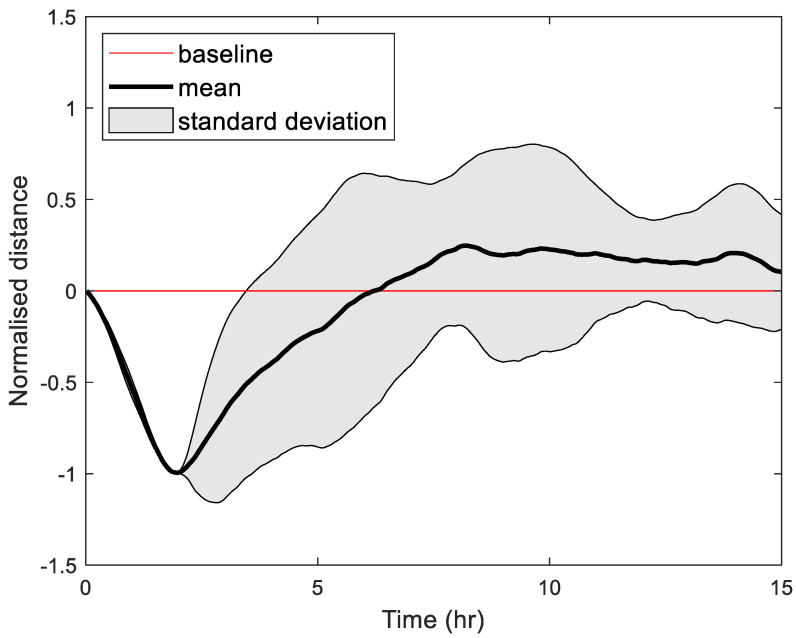
Normalised distance measurements for C58.

Hydration processes were monitored using infrared detection, and the normalised distance over 15 h is shown in Figure 6. An initial growth phase was observed during the first two hr, where the normalised distance decreased sharply, likely due to rapid water absorption causing expansion of the sample structure. This was followed by a transparency phase where a portion of the light goes through the sample rather than reflecting from its surface. Hence, the normalised distance steadily increased, peaking around 8 to 10 h, where the highest transparency appears to be achieved. Beyond this time point, the system approached equilibrium, with minimal changes in normalised distance observed after 12 h.

The shaded region indicates variability in response across the three samples. While the initial phase was consistent, variability increased during the initiation of the transparency phase of expansion, suggesting heterogeneity in sample behaviour regarding light reflection. By the 15 h mark, the normalised distance stabilised close to the baseline, indicating that steady transparency behaviour during hydration was achieved.

### 2.2. Discussion

Contaflex hydrogel materials were selected for this study due to their widespread usage and relevance in commercial soft contact lens manufacturing. Contaflex is a prominent representative of polymacon-based hydrogels extensively employed in contact lenses worldwide. Despite their popularity and commercial significance, detailed quantitative characterisation of hydration behaviour and swelling properties across a range of water-contents (38% to 77%) remains relatively limited. Given that different Contaflex variants exhibit varying degrees of hydrophilicity, comprehensively evaluating their hydration process is crucial for optimising lens designs and predicting their clinical performance.

In terms of the LEF, this analysis confirms that the Contaflex hydrogel samples exhibit stable swelling properties, closely aligning with the swelling performance anticipated by the manufacturer with minor deviations. However, this close alignment across multiple trials highlights the reproducibility and reliability of the material’s swelling behaviour.

Notably, swelling continued significantly beyond the manufacturer’s minimum hydration time of 15 h. Across all sample groups, measurable increases in LEF and REF were observed between 17 h and 24 h, particularly for higher water-content materials such as C67 and C77, indicating that hydration equilibrium had not yet been reached by 17 h. To capture this ongoing swelling phase, the primary analysis was extended to 24 h. While preliminary monitoring was conducted up to 72 h for a subset of samples, changes beyond 24 h were minimal (typically < 1%), indicating that most materials approached a stable hydration state by this point. The 24 h timeframe was therefore selected to balance experimental completeness with practical feasibility, capturing critical swelling behaviour while avoiding data redundancy.

A progressive increase in moisture uptake was observed, with C77 exhibiting the highest mass swelling (0.637 ± 0.134) and C38 the lowest (0.265 ± 0.128). These findings align with research on high water-content hydrogels, which often develop a hydration gradient between the front and back surfaces during wear. This gradient may contribute to uneven lens hydration and potential ocular complications such as corneal erosions, particularly in thinner hydrogel designs. Research highlights that higher water-content hydrogels, such as Gelfiex 60 (60% nominal water-content), often exhibit a hydration gradient between the front and back surfaces during wear, with the back surface typically being more hydrated. This gradient can potentially contribute to ocular complications such as corneal erosions, particularly in thin, high water-content lenses [6]. Jones et al. confirmed this hydrokintetic behaviour further in a comparative study of hydrogel and silicone hydrogel lenses, showing that dehydration rates were highest in materials with greater water-content [11].

Contaflex was hydrated in pH 7.1 buffered saline for 24 h, considering both practical and physiological factors. While the manufacturer recommends a minimum hydration period of 15 h in a pH 6.8 to 7.5 solution, no upper limit was specified, leaving room for optimisation based on experimental constraints and real-world conditions. Since contact lenses are ultimately intended for use on the human eye, selecting a hydration pH that closely mimics the tear film, typically around pH 7.1, was logical. This ensures that the material behaves as it would in vivo, minimising potential discrepancies between laboratory findings and clinical performance. Furthermore, the recommended second hydration step in pH 7.2 isotonic saline was omitted due to the necessity of maintaining precise alignment of cameras and light sensors throughout the experiment. Disrupting the hydration medium would have risked inconsistencies in data collection, ultimately impacting the reliability of the results. Extending hydration to 24 h and analysing results at 17 h as a slightly extended version of the manufacturer’s minimum recommendation ensured that both short-term and day-long hydration effects were captured, providing a more comprehensive understanding of the material’s behaviour over time.

The samples, referred to as C38, C55, C58, C67, and C77, with “C” indicating Contaflex, were named according to their manufacturer-reported water-contents: 38%, 55%, 58%, 67%, and 77%, respectively. Lab measurements, however, showed lower rounded values across the board: 27%, 33%, 52%, 61%, and 64%. On average, the lab-measured water-contents were about 11% lower than the values provided by the manufacturer. This difference may be due to variations in measurement methods, as the manufacturer’s exact protocol is not publicly available and may differ from the one used in this study.

The observed trend of decreasing refractive index with increasing water-content reflects the expected optical behaviour of hydrogel materials, where the incorporation of water (n ≈ 1.333) reduces the effective refractive index of the polymer matrix. The data supports the use of refractive index to provide a practical foundation for modelling lens parameters with soft contact lenses under physiological conditions.

Additionally, the water-content of hydrogel lenses is known to decrease with wear; a study on Bausch & Lomb lenses has demonstrated a reduction in water-content from 38.6% when new to 27.9% after wear, suggesting significant on-eye hydration changes over time [12]. Although this study does not evaluate the long-term dehydration behaviour of worn lenses, the observed swelling behaviour may differ when exposed to real-world conditions such as tear replenishment, fluid negative pressure, cyclic blinking dynamics, and eyelid interaction. This highlights the importance of considering on-eye hydration effects when designing contact lenses, as swelling observed in controlled environments may not fully translate to clinical performance.

The hydration behaviour of hydrogel lenses is further influenced by their interaction with chemical and environmental factors. Incubation in ophthalmic fluids, such as artificial tears or eye drops, has been shown to alter dehydration profiles and contact angles, suggesting modifications in water-content and overall hydration retention [9]. Similarly, prolonged exposure to hydrogen peroxide disinfection solutions significantly reduces water-content, though rehydration can be fully restored with appropriate neutralising solutions [13]. The pH of the precorneal tear film is also a critical determinant of hydrogel hydration, with higher pH levels associated with reduced water-content, which in turn impacts oxygen permeability and the overall fit of the lens [14]. These findings highlight the need to consider the broader chemical environment when assessing hydrogel swelling behaviour, particularly in clinical applications where exposure to varied ophthalmic solutions is routine.

Beyond external factors, the intrinsic chemical structure of the hydrogel material plays a fundamental role in hydration dynamics. Modifications such as incorporating a 2-methacryloyloxyethyl phosphorylcholine (MPC) polymer layer have enhanced hydrophilicity, reduced friction and improved hydration retention, which ultimately enhanced lens comfort during wear [15]. Advanced hydrogel designs integrating microchannel structures within poly(HEMA) matrices further promote capillary flow, supporting sustained hydration by facilitating moisture exchange during blinking [7]. These material innovations offer promising avenues for optimising lens performance, particularly in maintaining hydration stability over extended wear periods.

Although the Wilcoxon signed-rank test yielded a *p*-value of 0.0625 in both LEF and REF comparisons, which falls just above the conventional threshold for significance (*p* ≤ 0.05), the result is not interpreted as definitive evidence of no effect. Rather, it is considered suggestive of a trend. This interpretation is further supported by the Hodges–Lehmann estimators, which produced negative median differences with confidence intervals that did not cross zero, reinforcing the presence of a systematic effect.

The increased variability observed in the higher water-content groups is likely due to microstructural heterogeneity inherent to these materials. Hydrogels with higher equilibrium water-content are known to exhibit less uniform polymer network structures, which may lead to localised differences in swelling dynamics. Despite this, the overall swelling patterns remained consistent across replicates, suggesting that the findings are reproducible and reflect true material behaviour rather than experimental error.

The analysis also extends to the optical performance of various lens materials under a wide range of power targets. Simulation results reveal pronounced differences in power error trends across the lens designs, mainly driven by each material’s inherent properties. The C38 design, contrary to initial expectations, shows a steep and consistent increase in power error as target power rises, indicating that its material composition may be less stable than previously assumed. On the other hand, C55 and C77 exhibit more moderate, though still significant, linear error gradients. These increases in error with target power suggest that both materials could benefit from formulation or design geometry adjustments to improve accuracy and stability. Material C58 demonstrates the most stable behaviour, with only minimal variation across all tested powers, highlighting its potential as a reliable choice for precision applications. These findings are supported by literature indicating that mechanical properties like modulus of elasticity significantly impact lens shape retention and optical power under in vivo conditions [16]. Therefore, the material choice and design strategy should consider these aspects to minimise power deviations and enhance lens effectiveness [17,18]. It is important to note that the simulation was designed to focus specifically on dimensional changes during swelling. Clinical factors like blinking dynamics, tear surface tension, and eyelid pressure were not included as they fall outside the scope of this study. However, they were explored in earlier work [19,20,21].

The analysis of the LiDAR data provides valuable insights into the spatial and temporal variations within the dataset. The detailed measurements obtained from the LiDAR scans demonstrate high spatial resolution, enabling precise characterisation of surface profiles and structural changes over time. Notably, the consistency across repeated scans highlights the reliability of the system and its suitability for capturing dynamic processes.

In this study, a custom-built LiDAR system was employed to monitor transparency changes during the hydration process of hydrogel samples. LiDAR technology offers several advantages, including non-contact measurement, high spatial resolution, and capturing real-time data. While direct validation against traditional transparency measurement techniques was not conducted in this study, the application of LiDAR in materials research has been documented in the literature. LiDAR has been effectively used to measure glacier ice’s optical properties, demonstrating its ability to assess transparency and scattering characteristics with high precision. Additionally, LiDAR has been utilised to determine the surface shape of translucent objects by combining laser-based structured light with polarisation techniques, highlighting its versatility in characterising the optical properties of materials [22,23]. Given these established capabilities, the application of LiDAR in this study is justified by its potential to precisely track hydration-induced transparency changes in a sensitive and temporally resolved manner.

Designing a soft contact lens starts with defining the needed optical specs. Since the lens swells when hydrated, manufacturers use swell factors to estimate dry dimensions, but they can vary. Measuring them directly for each batch gives more accurate results. Once the final wet size was known, optical calculations were used to find the lens power, which was then compared to the target. This study used advanced MATLB simulations to show how differences between measured and reported swell factors affect final power.

However, some limitations in the data warrant discussion. The variability observed in certain dataset segments may reflect inconsistencies in surface reflectivity or environmental factors, such as lighting or atmospheric conditions, during the scans. These factors could introduce noise, particularly in regions with uneven surface geometry, or reflectance properties that vary. While preprocessing steps such as filtering and interpolation were applied to mitigate these effects, further refining these techniques may improve data quality.

One limitation of this study is the use of a single-step hydration protocol in pH 7.1 PBS, rather than the manufacturer’s recommended two-step pH adjustment (pH 8.2 to pH 7.2). This simplification was necessary to maintain alignment and continuity during optical and imaging measurements. However, it introduces the possibility that subtle differences in the swelling process or transparency behaviour may have occurred. Future work should directly compare these hydration methods to assess whether the simplified protocol yields equivalent material responses.

Another consideration is the challenge of aligning the LiDAR data with other experimental datasets, mainly when temporal or spatial scales differ. For example, discrepancies between LiDAR-derived metrics and parallel measurements, such as hydration or swelling data, may arise due to resolution or sampling frequency differences. The system captured reflected infrared signal intensity as a proxy for relative transparency changes across the sample surface. No direct calibration against UV-visible spectroscopy or refractometry was performed in this study; therefore, results are interpreted as relative indicators of change rather than absolute transmittance values. Future validation against spectrophotometric or refractometric methods is necessary to establish absolute accuracy and calibrate system sensitivity.

As presented in this study, the clinical implications of understanding hydrogel swelling behaviour are substantial, particularly regarding soft contact lens fitting accuracy, wearer comfort, and ocular health. Variations in LEF and REF expansion observed across Contaflex materials significantly affect lens geometry once hydrated, influencing optical power accuracy and lens fitting parameters. Accurate prediction of these parameters ensures optimal lens positioning and movement on the ocular surface, which is crucial for patient comfort, visual acuity, and safety.

Materials with higher water-content (such as C67 and mainly C77), demonstrating pronounced swelling and variability, may require more careful consideration and compensatory adjustments during the lens manufacturing process. Over- or under-estimating swelling could lead to lenses with incorrect sagittal depth, base curve, or diameter, potentially causing lens decentration, compromised optical performance, discomfort, or even adverse events like mechanical stress on the cornea and epithelial damage.

Furthermore, the demonstrated relationship between the hydration process and optical transparency highlights potential real-world impacts on lens clarity and visual quality. Ensuring lenses reach stable hydration and optimal transparency prior to dispensing to the patient can enhance wearer satisfaction and reduce complaints associated with initial visual blur or variable optical performance.

Clinicians should perhaps consider material-specific swelling characteristics reported here when prescribing and fitting lenses, particularly for lenses of higher refractive power or lenses intended for extended wear. Additionally, manufacturers could leverage these findings to refine their hydration protocols and lens designs, ultimately improving clinical outcomes for contact lens wearers.

## 3. Conclusions

This study examined the hydration behaviour of Contaflex soft contact lens materials, demonstrating that swelling dynamics vary with water-content. Higher water-content hydrogels exhibited greater expansion and hydration gradients, influencing fit and optical transparency. Significant differences in LEF and REF values were observed, particularly between C38 and C58 materials.

Integrating digital imaging and LiDAR provided precise tracking of hydration changes, confirming the role of environmental factors and material composition in swelling behaviour. These findings contribute to optimising hydrogel lens design for improved wearer comfort and ocular health.

Soft contact lens design simulation through a leading custom-built software tool highlighted how crucial material swelling is in designing soft contact lenses with accurate optical power. By comparing manufacturer-provided swell factors with actual measured values, it can be seen how swelling deviations impact the final lens power. The results show that while some materials, like C38, maintain consistent power accuracy, others, such as C55 and C77, experience systematic errors that require adjustments. These findings emphasise the importance of carefully refining lens design to ensure precise vision correction while staying within the ISO tolerance standards range.

## 4. Materials and Methods

**Figure 7:**
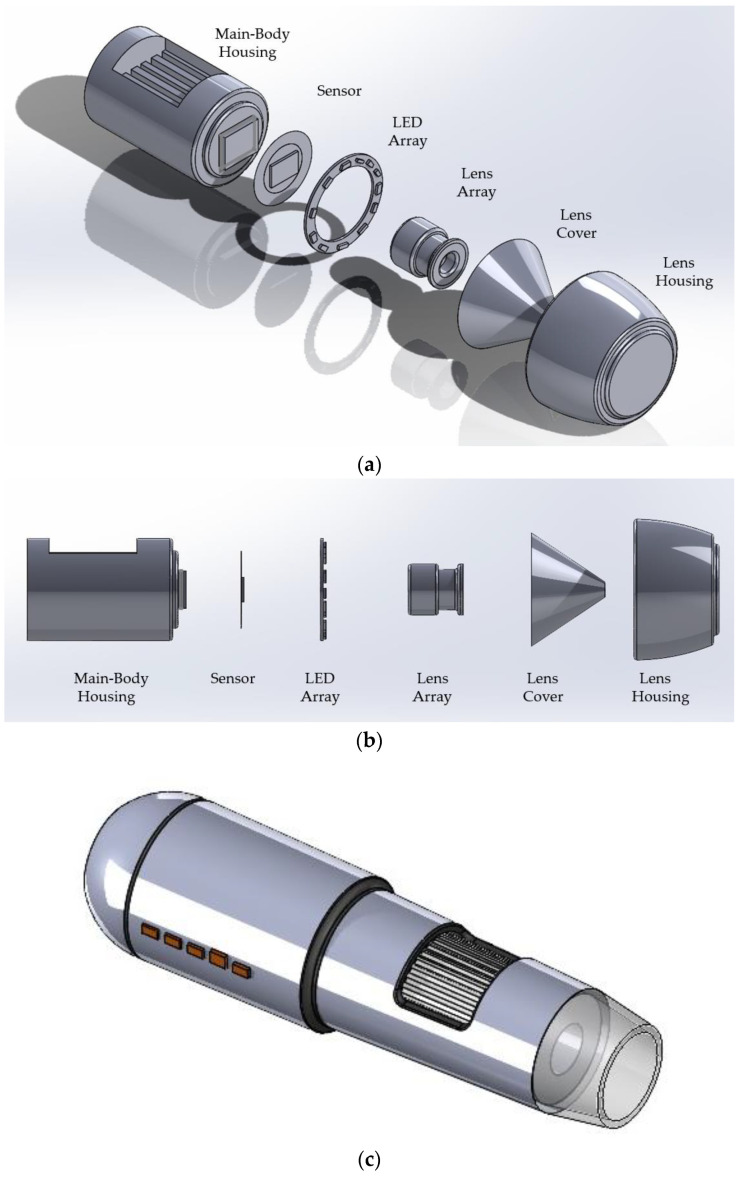
(**a**) Isometric exploded view of Jiusion camera, (**b**) Side exploded view of a Jiusion camera, (**c**) Three-dimensional view of the Jiusion camera.

Two 2560 × 1440 pixels high-resolution microscopic cameras (Jiusion, Shenzen, China) were employed to monitor the growth of hydrogel samples. An ‘exploded’ view of the cameras utilised can be seen in Figure 7. One camera was positioned to capture the diameter growth from a bottom-up view, while the other camera focused on the height growth from a side view.

The hydrogel samples were placed in a 120 mm triple vent square plastic Petri dish filled with PBS solution and suspended on an 80 mm ring stand to ensure stability and optimal viewing angles for both cameras. The hydrogel was placed on a 5 mm square piece of sandpaper to prevent drifting. It was ensured that the Petri dish’s surface did not block the sample’s surfaces to allow the flow of PBS solution. The ring stand and cameras were levelled with a bullseye bubble spirit 65 mm level to prevent any further drift of the hydrogel in the solution, maintaining the focus and position of the samples throughout the observation period.

**Figure 8:**
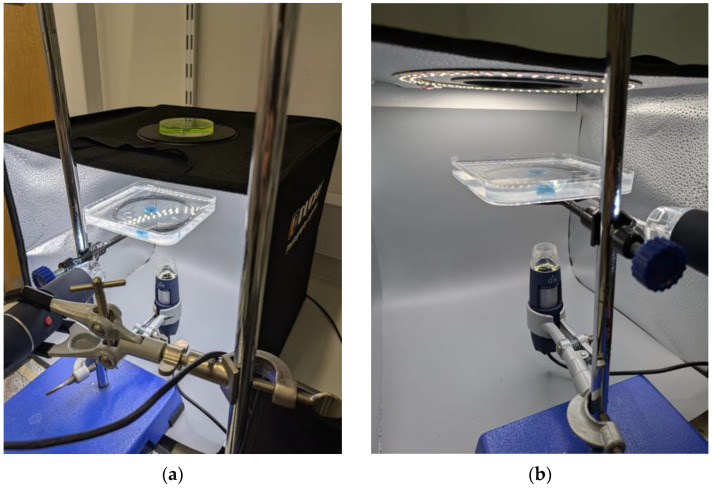
Duclus photography light box with sample in situ, (**a**) Right angle view, (**b**) Left angle view.

**Figure 9:**
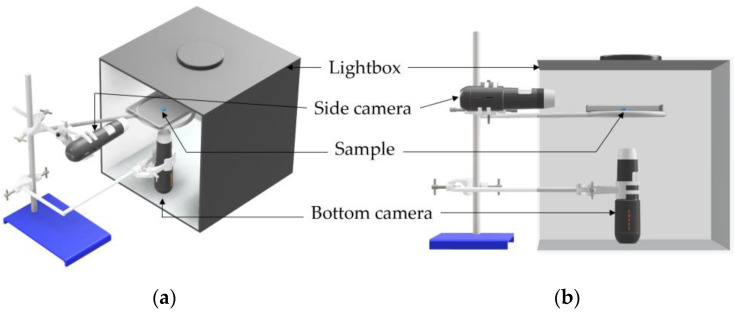
CAD representation of the experimental setup, (**a**) isometric, (**b**) side view.

The experimental setup was situated within a photography light box (Duclus, Seoul, Republic of Korea), Figure 8, to provide sufficient and consistent lighting, ensuring that high-quality images were captured. This controlled lighting environment minimised shadows and reflections, enhancing the clarity and detail of the images. Figure 9 shows a computer-aided design (CAD) representation of the experimental setup.

The tested material was Contaflex, with 38%, 55%, 58%, 67% and 77% nominal water-contents as reported by Contamac LTD, five samples each. For ease of reference throughout the manuscript, these groups are denoted as C38, C55, C58, C67, and C77, respectively, corresponding to their supposed water-content percentages. Contaflex material should be hydrated in a pH 6.8 to 7.5 buffered saline solution for a minimum of 15 h in a controlled environment at 20 ± 2 °C. However, no maximum hydration time was specified. The manufacturer’s protocol recommends two steps; between them, the hydrated lenses are transferred from pH 8.2 to a pH 7.2 solution for 2 h. Following this two-step protocol was not feasible in the present study, as changing the hydration fluid would have disrupted the experimental setup, particularly the alignment of cameras and LiDAR sensors, which was critical for data consistency. In order to maintain compliance with the manufacturer’s general hydration recommendations while ensuring experimental integrity, the lenses were hydrated in buffered saline at pH 7.1. This pH was chosen to approximate the average pH of human tears [24], reflecting conditions under which soft contact lenses would be used in clinical settings.

Hydration was conducted for 24 h, with measurements taken at 17 h, slightly exceeding the recommended 15 h minimum, and at 24 h to represent a full day. The pH of the PBS was verified for all samples before each test using a calibrated pH meter (Hanna Instruments, Leighton Buzzard, Bedfordshire, UK).

### 4.1. High-Resolution Digital Image Tracking System

An in-house custom-built Python 3.12.4 (Python Software Foundation, Wilmington, DE, USA) scripts were written to automate the image-capturing process, automatically trigger the camera, capture an image every minute, and save it to a local folder on the computer. The source code for this operation can be found in Appendix A. The images were captured in 2K resolution, 2560 by 1440 pixels. Images were captured across a 24 h period. Captured images were automatically saved with timestamps to maintain chronological order and organised into separate directories for bottom and side views.

The collected images were compiled into a timelapse video using another custom-built Python script. Images were sequentially ordered based on their timestamps, and the ordered images were processed and rendered into a 2K timelapse video in an automated process. The source code for this operation can be found in Appendix B. This provided a clear and continuous view of the hydrogel’s height and diameter growth over time. The timelapse video was then used to further analyse the hydrogel’s growth patterns and swelling dynamics. The fully automated hydration monitoring process used in the study allowed for systematic, repeatable examination of hydrogel growth, providing perceptions of the material’s properties and behaviour through analyses.

The LEF and REF were calculated to quantify dimensional changes during hydration. LEF was defined as the ratio of the hydrogel’s swollen dimension to its dry dimension in the linear direction, while REF was defined as the ratio of swollen to dry dimensions in the radial direction (Equation (1)). These values were derived from calibrated image sequences captured by the side-view and bottom-view cameras.

(1) $$LEF/REF=\frac{wet\;dimension\;(post-hydration)}{dry\;dimension\;(pre-hydration)}in\;(linear/radial)\;direction$$

High-resolution images were calibrated into focus with a scale that showed precise sub-millimetre readings in the first frame of the capture. These markings were then used to calibrate the distance interpretation in a custom-built high-resolution digital image-tracking software system, “Tracker Video Analysis and Modelling Tool” (Version 6.0.2). Hydrated dimensions were obtained at the 17 h and 24 h timepoints by tracking the position of opposing edge points using the tracker software. This process enabled consistent, frame-accurate extraction of swelling metrics across all samples. Sample dimensions were measured using a digital Vernier calliper (D00352, Duratool, Taichung, Taiwan) with 10 µm accuracy before and after hydration to allow for accurate calibration of tracking parameters.

### 4.2. Transparency Measurement via Light Detection and Ranging System

An analysis of the transparency of the C58 hydrogel throughout hydration was conducted by employing a LiDAR sensor. The Adafruit VL6180X Time of Flight Micro-LiDAR Distance Sensor Breakout (STM Electronics, Geneva, Switzerland) was selected due to its ability to function well at close range, which was imperative due to the confined conditions of the experiment setup. This sensor was paired with an Arduino MEGA2560 (Arduino, Somerville, MA, USA) microcontroller. The choice of Arduino MEGA2560 for running micro-LiDAR sensors was due to its high number of digital and analogue pins, ample memory, and multiple serial communication ports, enabling simultaneous integration of LiDAR modules and other peripherals. MEGA2560’s reliable performance and broad community support simplify the development and debugging processes. When conducting the analysis, the LiDAR sensor was positioned 25 mm above the hydrogel sample. Optimal levelling was ensured throughout to minimise the effects of refraction. This was validated by a high-precision multi-axis spirit level (Taskar, Sale, UK) with an accuracy of ±0.2°. To ascertain the correct positioning of the LiDAR sensor, the bottom-view camera used to track the diameter was utilised as it provided a frame of vision that included both the hydrogel and the LiDAR sensor, allowing for extreme precision when performing alignment. Following a custom-written code for Arduino IDE 1.8.19, the sensor transiently measures the distance between itself and the hydrogel, automatically taking a reading every second for the 24 h tracking timeframe. Although LiDAR measurements were recorded continuously over one day, only the first 15 h are presented in the results, as minimal changes in transparency were observed beyond this point.

**Figure 10:**
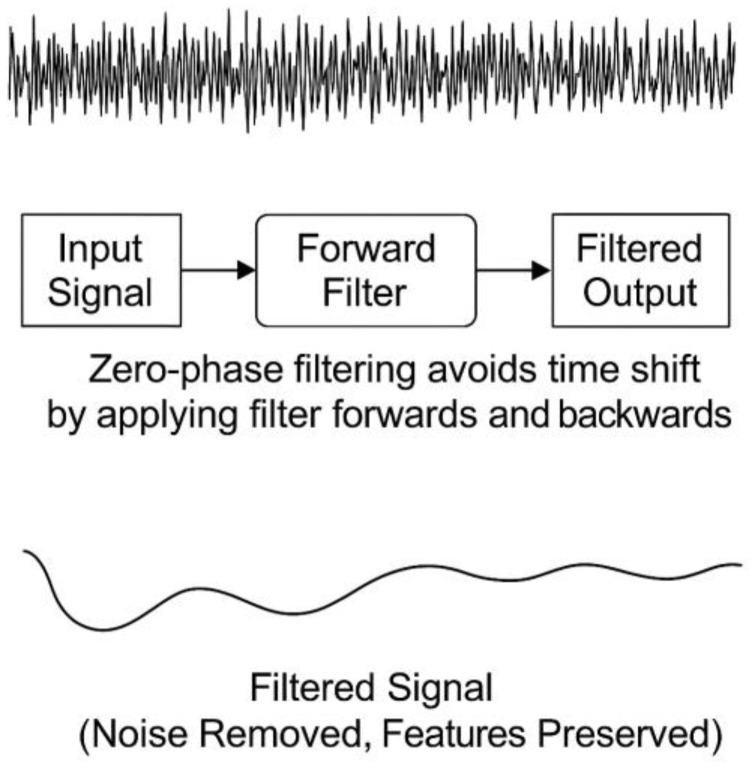
Zero-phase shift filtering processing.

Upon collecting the data from a trial, the signal digital noise was cut down using a custom-built lowpass filter function, ensuring zero phase shift, using MATLAB R2024b Signal Processing Toolbox (Mathworks, Natick, MA, USA). In this digital signal processing context, zero-phase shift filtering refers to a filtering approach that eliminates the time shift, also known as phase distortion, typically introduced by conventional filters [25]. Unlike traditional forward filters that delay signals and shift their features in time, zero-phase filters apply forward and backwards filters across the data [26]. This filtering approach attenuated high-frequency noise components, preserving essential signal features for subsequent analysis (Figure 10).

### 4.3. Refractive Index

A laser-based optical refraction setup was employed to determine the refractive index of hydrated hydrogel buttons. A Huepar AG01 Digital Angle Gauge with an angular accuracy of ±0.01° was used to project a stable, collimated red laser beam (Quarton VLM-635-32 LPA, 635 nm, CW) at a fixed incident angle of 60° from the surface normal. The laser was mounted on a calibrated rotary stage to ensure precise alignment.

**Figure 11:**
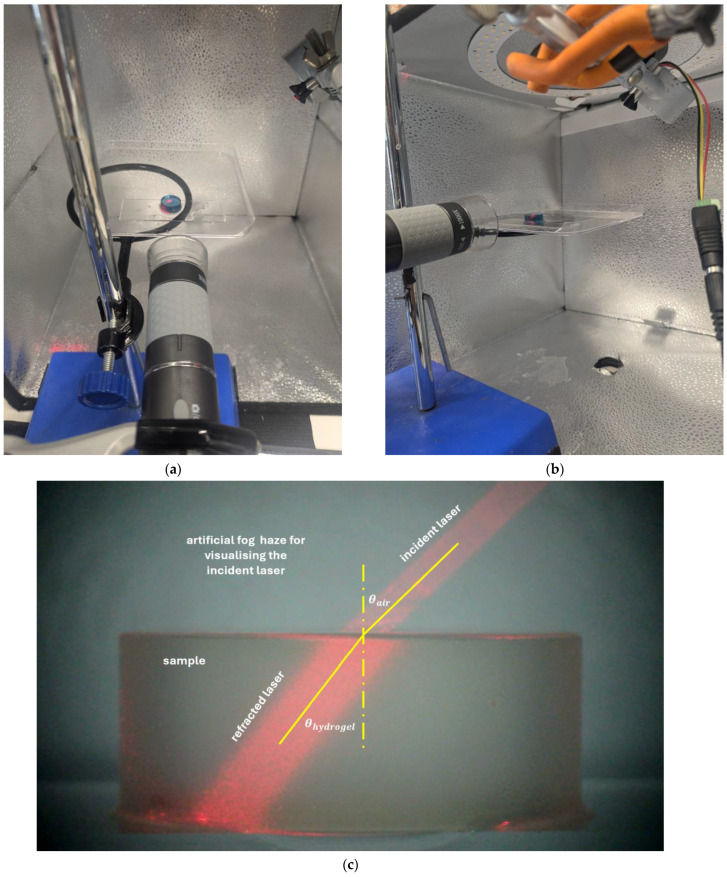
(**a**) Refractive index measurement setup where a laser was used to determine the refractive effect of hydrogel on light, (**b**) Angle view of the same setup, (**c**) A photo of a sample was taken for display purposes, with an incident angle of 45° instead of 60° to improve visualisation. Ozone-safe fog spray visualised the incident laser ray better in this photo, but was not used during measurements.

Each hydrogel button was placed on a standard borosilicate glass coverslip, ensuring flat and uniform contact. The laser beam entered the hydrogel from the air at a known angle and exited into the underlying glass substrate. The beam path was imaged orthogonally using a Jiusion 4K USB microscope camera, capturing the internal refraction angle (Figure 11).

High-resolution images were analysed using ImageJ (version 1.54p), an open-source platform for biological image analysis [27,28]. The angle gauge was used to measure the refracted beam path. The internal refraction angle within the hydrogel was calculated, and the hydrogel’s refractive index was determined by applying Snell’s law [29]:

(2) $$n_{hydrogel}=\frac{n_{air}\sin\theta_{air}}{\sin\theta_{hydrogel}}$$

where the $\theta_{air}$ is the incident laser angle in the air (set to 60°), $\theta_{hydrogel}$ the refracted laser angle in the hydrogel and the refractive index of air is $n_{air}=1$. Internal reflection and beam distortion within the sample were visually assessed and found to be negligible.

### 4.4. Statistical Analysis

Statistical techniques were conducted to evaluate the significance of differences in LEF & REF values between time points and across sample groups.

The Wilcoxon signed-rank test was selected as an appropriate statistical method for comparing the 17 h and 24 h measurements within each sample group due to its suitability for small sample sizes and its ability to analyse paired data without assuming normality. Unlike parametric tests such as the paired t-test, which requires the differences between paired observations to be normally distributed, the Wilcoxon test is a non-parametric alternative that evaluates whether the median difference between paired observations significantly deviates from zero. Given that each sample group in this study consists of only five observations, assessing normality is impractical, making non-parametric methods more appropriate.

The Wilcoxon signed-rank test has been widely applied in biomedical and materials science research where sample sizes are limited, and the underlying data distribution is unknown. The advantages of non-parametric tests in handling small, non-normally distributed datasets highlight their robustness compared to parametric alternatives. This study demonstrates that non-parametric methods, such as the Wilcoxon test for sample sizes as low as five, provide reliable statistical inference while minimising the risk of violating normality assumptions [30].

The HL estimator was employed in this study to robustly estimate the median difference between 17 h and 24 h measurements. As a non-parametric measure, it is particularly suitable for small sample sizes and does not rely on assumptions of normality, enhancing its robustness against outliers. This estimator calculates the median of all possible pairwise averages of differences, providing a reliable measure of central tendency even in limited data scenarios. Its application is well-documented in non-parametric statistical methods [31].

### 4.5. Soft Contact Lens Geometry Simulation

**Figure 12:**
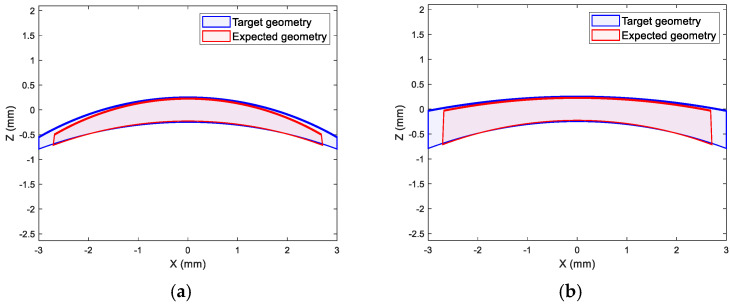
The geometry of the centre optic zones of soft contact lenses of (**a**) 20 D and (**b**) −20 D target powers, in blue, against the expected geometry when the measured swell factor of C77 was applied, in red. Wet central thickness was set to a relatively high nominal value of 0.5 mm in this example to improve visualisation.

In this simulation, the soft contact lens geometry formation began by selecting a back surface curve of 8.6 mm, as a classic commonly used value, and setting a target power to the range of −20 D to 20 D. Figure 12 shows optic zones of ±20 D target power lenses as examples where the front surface curvatures were calculated to achieve the desired power following [19,20,32] based on the manufacturer refractive index. As a case, the manufacturer’s swell factor of C77 was applied to convert the nominal dimensions from wet to their nominal dry state, followed by the use of the lab-measured swell factor reversely to determine the expected wet dimensions. The front and back surfaces were fitted to circles to calculate their effective central curvatures. The anticipated power of the lens was determined and compared with the target power using these curvatures following the methods described in [16,19,20] and the lab-measured refractive index. Finally, the error was calculated to assess the deviation between the achieved and intended power, Figure 12.

## Figures and Tables

**Table 1:** Mean and variance for all LEF and REF groups.

Contaflex Material Group Based on Their Nominal Water-Content	LEF (17 h)	LEF (24 h)	REF (17 h)	REF (24 h)	Manufacturer Swell Factor
	Mean ± Std	Mean ± Std	Mean ± Std	Mean ± Std	
C38	1.182 ± 0.019	1.201 ± 0.019	1.154 ± 0.010	1.179 ± 0.011	1.20
C55	1.206 ± 0.067	1.218 ± 0.070	1.180 ± 0.067	1.209 ± 0.080	1.35
C58	1.290 ± 0.047	1.353 ± 0.011	1.325 ± 0.039	1.353 ± 0.042	1.36
C67	1.428 ± 0.027	1.452 ± 0.017	1.337 ± 0.052	1.389 ± 0.055	1.47
C77	1.499 ± 0.045	1.563 ± 0.093	1.485 ± 0.053	1.536 ± 0.052	1.66

**Table 2:** Statistical parameters for LEF testing comparing 17 h and 24 h. HL stands for the Hodges–Lehmann estimator.

Group	LEF HL Median	LEF 95% CI Lower	LEF 95% CI Upper
C38	−995.94	−1002.42	−982.56
C55	−1044.28	−1044.39	−978.44
C58	−1038.75	−1117.89	−1037.15
C67	−1179.62	−1250.74	−1179.62
C77	−1216.7	−1223.16	−1216.7

**Table 3:** Statistical values for REF testing comparing 17 h and 24 h. HL stands for the Hodges–Lehmann estimator.

Group	REF HL Median	REF 95% CI Lower	REF 95% CI Upper
C38	−956.76	−967.9	−955.66
C55	−962.64	−1060.4	−952.55
C58	−1072.23	−1173.68	−1072.23
C67	−1060.4	−1132.43	−1060.4
C77	−1181.54	−1277.85	−1181.54

**Table 4:** Refractive index (*n*) of raw Contaflex lenses measured at 17 h and 24 h hydration time points.

	n (17 h)	n (24 h)	Manufacturer Published Value
Group	Mean ± Std	Mean ± Std	
C38	1.552 ± 0.063	1.526 ± 0.097	1.44
C55	1.461 ± 0.043	1.418 ± 0.427	1.41
C58	1.417 ± 0.036	1.354 ± 0.409	1.4
C67	1.399 ± 0.028	1.388 ± 0.439	1.39
C77	1.414 ± 0.070	1.372 ± 0.458	1.38

**Table 5:** Mass swelling ratios of Contaflex hydrogels after 24 h of hydration. M_1_ = swollen mass; M_0_ = dry mass.

Sample Group	Lab Measured Water-Content (%) $\frac{M_{1}-M_{0}}{M_{1}}\times 100$	Manufacturer Published Nominal Water-Content (%) $\frac{M_{1}-M_{0}}{M_{1}}\times 100$
C38	26.5 ± 12.8	38
C55	33.1 ± 15.6	55
C58	52.2 ± 5.3	58
C67	60.7 ± 2.4	67
C77	63.7 ± 13.4	77

## Data Availability

The data presented in this study are available on request from the corresponding author. Not all data are publicly available due to considerations regarding possible future commercialisation.

## Abbreviations

The following abbreviations are used in this manuscript:

CAD	Computer-aided design
CC	Contamac contaflex
CI	confidence intervals
HL	Hodges–Lehmann
ISO	International Organisation for Standardisation
LEF	Linear expansion factor
LiDAR	Light detection and ranging
OCT	Optical coherence tomography
PBS	Phosphate-buffered saline
pHEMA	Poly hydroxyethyl methacrylate
REF	Radial expansion factor

## Appendix A

The Python custom-built code used for timelapsing in the current study is represented in this Appendix. Although this article is published under a CC BY open-access licence, the Python custom-built code presented in Appendix A is not covered by this CC BY licence. The code is shared solely for review and reproducibility purposes. Any use beyond this scope, including reuse, modification, or distribution, requires prior written permission from the corresponding author.

The script uses the open-source computer vision (OpenCV) to capture images from the Jiusion camera at a specified time interval (in seconds). Each image is overlayed with a high precision timestamp (to the millisecond), and is sequentially saved in a specified output folder. The image stream is displayed in a window, and the process continues until the user presses the ‘q’ key. Images are captured at maximal quality (3840 × 2160 pixels).

import cv2, os, time from datetime import datetime def capture_images_from_webcam(output_folder, interval): while True: try: if not os.path.exists(output_folder): os.makedirs(output_folder) cap = cv2.VideoCapture(0) if not cap.isOpened(): time.sleep(interval) continue cap.set(cv2.CAP_PROP_FRAME_WIDTH, 3840) cap.set(cv2.CAP_PROP_FRAME_HEIGHT, 2160) width = int(cap.get(cv2.CAP_PROP_FRAME_WIDTH)) height = int(cap.get(cv2.CAP_PROP_FRAME_HEIGHT)) img_counter = 0 while True: ret, frame = cap.read() if not ret: raise Exception(“Capture failed.”) timestamp = datetime.now().strftime(“%Y-%m-%d %H:%M:%S.%f”)[:-3] font = cv2.FONT_HERSHEY_SIMPLEX scale, colour, thick = 2, (0, 255, 255), 3 sz, _ = cv2.getTextSize(timestamp, font, scale, thick) pos = (width - sz[0] - 10, height - 10) cv2.putText(frame, timestamp, pos, font, scale, colour, thick, cv2.LINE_AA) img_path = os.path.join(output_folder, f”image_{img_counter:04d}.png”) cv2.imwrite(img_path, frame) img_counter += 1 cv2.imshow(‘Webcam’, frame) t0 = time.time() while time.time() - t0 < interval: if cv2.waitKey(1) & 0xFF == ord(‘q’): cap.release() cv2.destroyAllWindows() return except Exception: time.sleep(interval) # Parameters output_folder = r” interval = 60 capture_images_from_webcam(output_folder, interval)

## Appendix B

The Python custom-built code used for video compounding in the current study is represented in this Appendix. Although this article is published under a CC BY open-access licence, the Python custom-built code presented in Appendix B is not covered by this CC BY licence. The code is shared solely for review and reproducibility purposes. Any use beyond this scope, including reuse, modification, or distribution, requires prior written permission from the corresponding author.

This Python script generates a timelapse video from a sequence of images stored in a target folder post test. It uses OpenCV to read and compile the images into a video file at a user-defined frame rate (fps) and codec setting. Images are sorted alphabetically by filename to preserve temporal order.

import cv2 import os def create_timelapse(input_folder, output_file, fps): # Verify if the input folder exists if not os.path.exists(input_folder): print(f”Error: The directory {input_folder} does not exist.”) return # Get list of image files in the folder images = [img for img in os.listdir(input_folder) if img.endswith(“.jpg”) or img.endswith(“.png”)] if not images: print(f“Error: No image files found in the directory {input_folder}.”) return images.sort() # Sort the images by filename # Read the first image to get the frame size first_image_path = os.path.join(input_folder, images[15]) frame = cv2.imread(first_image_path) if frame is None: print(f“Error: Unable to read the first image file {first_image_path}.”) return height, width, layers = frame.shape # Initialise the video writer fourcc = cv2.VideoWriter_fourcc(*’XVID’) video = cv2.VideoWriter(output_file, fourcc, fps, (width, height)) # Add each image to the video for image in images: image_path = os.path.join(input_folder, image) frame = cv2.imread(image_path) if frame is None: print(f“Warning: Unable to read the image file {image_path}. Skipping.”) continue video.write(frame) # Release the video writer video.release() print(f“Timelapse video saved as {output_file}”) # Parameters input_folder = r” # Replace with your folder path output_file =” # Output video file name fps = 30 # Frames per second # Print the input folder path to verify it print(f“Using input folder: {input_folder}”) # Create the timelapse video create_timelapse(input_folder, output_file, fps)
